# PI3K/Akt-Independent NOS/HO Activation Accounts for the Facilitatory Effect of Nicotine on Acetylcholine Renal Vasodilations: Modulation by Ovarian Hormones

**DOI:** 10.1371/journal.pone.0095079

**Published:** 2014-04-14

**Authors:** Eman Y. Gohar, Sahar M. El-gowilly, Hanan M. El-Gowelli, Maha A. El-Demellawy, Mahmoud M. El-Mas

**Affiliations:** 1 Department of Pharmacology and Toxicology, Faculty of Pharmacy, Alexandria University, Alexandria, Egypt; 2 Medical Biotechnology Department, City for Scientific Research & Technology Applications, Borg El-Arab, Alexandria, Egypt; Medical Faculty, Otto-von-Guericke University Magdeburg, Medical Faculty, Germany

## Abstract

We investigated the effect of chronic nicotine on cholinergically-mediated renal vasodilations in female rats and its modulation by the nitric oxide synthase (NOS)/heme oxygenase (HO) pathways. Dose-vasodilatory response curves of acetylcholine (0.01–2.43 nmol) were established in isolated phenylephrine-preconstricted perfused kidneys obtained from rats treated with or without nicotine (0.5–4.0 mg/kg/day, 2 weeks). Acetylcholine vasodilations were potentiated by low nicotine doses (0.5 and 1 mg/kg/day) in contrast to no effect for higher doses (2 and 4 mg/kg/day). The facilitatory effect of nicotine was acetylcholine specific because it was not observed with other vasodilators such as 5′-*N*-ethylcarboxamidoadenosine (NECA, adenosine receptor agonist) or papaverine. Increases in NOS and HO-1 activities appear to mediate the nicotine-evoked enhancement of acetylcholine vasodilation because the latter was compromised after pharmacologic inhibition of NOS (L-NAME) or HO-1 (zinc protoporphyrin, ZnPP). The renal protein expression of phosphorylated Akt was not affected by nicotine. We also show that the presence of the two ovarian hormones is necessary for the nicotine augmentation of acetylcholine vasodilations to manifest because nicotine facilitation was lost in kidneys of ovariectomized (OVX) and restored after combined, but not individual, supplementation with medroxyprogesterone acetate (MPA) and estrogen (E2). Together, the data suggests that chronic nicotine potentiates acetylcholine renal vasodilation in female rats via, at least partly, Akt-independent HO-1 upregulation. The facilitatory effect of nicotine is dose dependent and requires the presence of the two ovarian hormones.

## Introduction

The NOS/NO and HO/CO pathways play important roles in vascular control. In the kidney, NO vasodilates the glomerular vasculature, decreases glomerular capillary pressure and prevent renal inflammatory injury [1], [2]. Other vascular effects of NO include the inhibition of neutrophil aggregation, adhesion of leukocytes to endothelium, and proliferation of vascular smooth muscle [3], [4]. Moreover, the NOS-derived NO plays a fundamental role in acetylcholine-mediated vasodilations in renal vasculature [5]. Alternatively, heme oxygenase (HO) is a microsomal enzyme that participates in heme breakdown to generate carbon monoxide (CO) [6]. Both constitutive (HO-2) and inducible (HO-1) isoforms of HO are expressed in renovascular structures [7]. HO-2 exerts a tonic regulatory effect on renal function. While the basal levels of expression of HO-1 in the rat kidney are relatively low, its contribution to HO activity becomes more apparent under pathophysiological conditions causing HO induction [8]. The HO-derived CO has been also implicated in the vasodilatory effect of acetylcholine in some vascular beds [9], [10].

Nicotine, a key component of the cigarette smoke, has been largely implicated in the smoking-induced vascular changes. Nicotine exerts widely contrasting vascular effects including vasodilation and vasoconstriction. The vasodilator effect of nicotine has been demonstrated in some vascular beds including renal arteries [11]–[13]. The mechanisms of nicotine-induced vasodilation are attributed to the activation of eNOS [11], cyclooxygenase [14], neuronally released calcitonin gene-related peptide [15], or nitric oxide released from perivascular nitrergic nerve terminals [16]. Nicotine also cause renal vasoconstriction and damage probably via increasing levels of vasoconstrictors, such as catecholamines, vasopressin and endothelin-1 [17]–[19]. Further, nicotine impairs NOS-dependent [20] and β-adrenoceptor vasoreactivity [21], [22] These discrepant vascular effects of nicotine could be attributed to differences in vascular beds or in the dose or duration of nicotine regimens.

It is worth mentioning that most of the published work on the vascular effects of nicotine has been conducted in the male population [11], [17], [21] or in pooled populations from both genders [16]–[19]. We have recently demonstrated that estrogen enhances the vasodilator effect of acute nicotine in the renal vasculature of female rats mainly through facilitation of NOS signaling [12]. Alternatively, acute nicotine attenuates isoprenaline vasodilations in the female renal vascular bed, an effect that is downregulated by the NO/cGMP signaling [22]. In a more recent study, we reported on the chronic effect of nicotine on renal vasodilations caused by adenosine receptor activation (NECA) and the modulation of this interaction by ovarian hormones [23]. The treatment of OVX rats with E2 or MPA enhances NECA vasodilation and this effect is intensified or depressed, respectively, upon nicotine administration [23]. The goal of this study was twofold. First, to determine whether nicotine acts in a similar hormonally-dependent fashion to alter the vasodilatory action of acetylcholine in the female renal vasculature. Second, and more importantly, to test the hypothesis that gaseous products of NOS (NO) and/or HO (CO) mediate the nicotine-acetylcholine renovascular interaction. In-vitro studies were undertaken in the rat isolated perfused kidney preconstricted with phenylephrine to assess the effect of (i) pharmacologic modulators of NOS and HO activities on the interaction of nicotine with acetylcholine responses, and (ii) depletion and repletion of ovarian hormones on the nicotine-acetylcholine interaction.

## Materials and Methods

### Drugs

Nicotine (Merck Schuchardt OHG, Hohenbrunn, Germany), acetylcholine chloride, hemin, N^w^-nitro-L-arginine methylester hydrochloride (L-NAME), L-arginine hydrochloride, papaverine hydrochloride, mifepristone, phenylephrine hydrochloride, N-ethylcarboxamidoadenosine, 17β-estradiol, zinc protoporphyrin IX (ZnPP, Sigma Chemical Co., St. Louis, MO, U.S.A.), medroxyprogesterone acetate (Pfizer, New York, U.S.A.), thiopental sodium (Thiopental, Biochemie GmbH, Vienna, Austria) were purchased from commercial vendors.

### Animals

Female Wistar rats (180–200 g, 20–22 weeks old, Animal Facility of the Faculty of Pharmacy, Alexandria University, Egypt) were used. All experiments were approved by the institutional Animal Care and Use Committee (IACUC, Faculty of Pharmacy, Alexandria University, Egypt, Permit Number 10–45). Surgery was performed under thiopental anesthesia, and all efforts were made to minimize suffering.

### The rat isolated perfused kidney

The rat kidney was isolated and perfused according to the method described in our previous studie [24], [25]. Briefly, rats were anesthetized with thiopental sodium (50 mg/kg, i.p.), the abdomen was opened by a midline incision and the left kidney was exposed. The left renal artery was dissected free from its surrounding tissues. Loose ties were made around the renal artery and the abdominal aorta, proximal and distal to the renal artery. A beveled 18-gauge needle connected to a 5-ml syringe filled with heparinized saline (100 U/ml) was used for cannulation. The aorta was ligated, and the left renal artery was cannulated via an incision made in the aorta. The cannula was immediately secured with ligatures and the kidney was flushed with heparinized saline and rapidly excised from its surrounding tissues. The kidney was transferred into a jacketed glass chamber maintained at 37°C and continuously perfused with Krebs' solution (NaCl 120, KCl 5, CaCl_2_ 2.5, MgSO_4_.7H2O 1.2, KH_2_PO_4_ 1.2, NaHCO_3_ 25, and glucose 11 mM) maintained at 37°C and gassed with 95% O2 and 5% CO_2_. Kidney perfusion was adjusted at a constant flow rate of 5 ml/min using a peristaltic pump (Model P3-Pharmacia Fine Chemicals). The kidney perfusion pressure was continuously monitored by means of a blood pressure transducer (Model P23XL, Astro-Med, Inc., West Warwick, RI, USA) distal to the pump and attached through MLAC11 Grass adapter cable to a computerized data acquisition system with LabChart-7 pro software (Power Lab 4/35, model ML866/P, AD Instruments, Bella Vista, Australia). An equilibration period of 30 min was allowed at the beginning of the experiment to ensure stabilization of the kidney perfusion pressure. To study the vasodilatory effects of acetylcholine, NECA, or papaverine, the renal vascular tone was elevated by continuous infusion of the α_1_-adrenoceptor agonist phenylephrine (10 µM) as described in our previous studies [24], [25].

### Ovariectomy

Bilateral ovariectomy was performed as described in our previous studies [26], [27]. A single 2–3 cm incision was made in the back and ovaries were isolated, tied-off with sterile suture and removed. Sham operation involved exposure of the ovaries without isolation. Each rat received i.m. injection of 50,000 U/kg of procaine penicillin (Procaine Penicillin-fortified, Chemical Industries Development Co., Egypt). Rats were housed individually in separate cages and allowed 2 weeks to recover prior to experimentation in perfused kidneys.

### Western blotting

The kidney was homogenized on ice in a homogenization buffer [50 mM Tris (pH 7.5), 0.1 mM EGTA, 0.1 mM EDTA, 2 µM leupeptin, 1 mM phenylmethylsulfonyl fluoride, 0.1% (vol/vol) Nonidet P-40, 0.1% SDS, and 0.1% deoxycholate]. After centrifugation (12,000 g for 10 min), protein in the supernatant was quantified (BCA protein assay kit, Pierce, USA). Protein extracts (10–15 µg per lane) were run on a 4–12% SDS-PAGE gel (Precast gradient uniform ready-made gels, Pierce, USA) and electroblotted to nitrocellulose membranes. Blots were blocked for 60 min at room temperature in Tris buffer saline (TBS; 10 mM Tris, 150 mM NaCl) containing 5% non-fat milk. They were then incubated for 60 min in TBS containing 0.01% non-fat milk with rabbit antibody to rat heme oxygenase (1∶1000, Pierce, USA), p-Akt (Thr 308, 1∶1000, Pierce, USA), Akt (1∶750, Sigma, USA), or β-actin (1∶1000, Pierce, USA). After 4 washes with TBS/0.05% Tween 20, blots were incubated for 30 min at room temperature with a goat anti-rabbit IgG HRP conjugated secondary antibody (Pierce, USA). After 5 washes with TBS, blots were detected by nitro-blue tetrazolium/5-bromo-4-chloro-3′-indolyphosphate (NBT/BCIP) insoluble color substrate. Bands were quantified by measuring the integrated density (mean density x area) using TotalLab Quant software (North Carolina, USA). Data were normalized in relation to β-actin and expressed as a percent of control values [28]–[30].

### Protocols and experimental groups

#### Dose-effect relationship of chronic nicotine on renal vasodilations

A total of 5 groups (n = 6–8 each) of sham-operated rats were employed in this experiment to investigate the effect of chronic administration of nicotine (0.5, 1, 2, or 4 mg/kg/day i.p. for 2 weeks) or equal volume of saline on the renal vasodilatory responses elicited by acetylcholine. To test for the specificity of the nicotine-acetylcholine interaction, vasodilatory responses to NECA, an adenosine analogue, or papaverine, a phosphodiesterase inhibitor, were also established in the same rats. On the experiment day, the kidneys were isolated from thiopental (50 mg/kg)-anesthetized rats, perfused, and constricted with continuous infusion of phenylephrine as described above. The renal vasodilatory responses to cumulative bolus doses of acetylcholine (0.01–2.43 nmol), NECA (1.6–50 nmol), and papaverine (1–243 nmol) were established and changes in the renal perfusion pressure were computed. Generally, a given dose of each vasodilator was injected when the vasodilatory response to the previous dose reached its peak. The right kidneys were isolated, immediately frozen in liquid nitrogen, and stored at −80°C for determination of the protein expression of HO-1 and phosphorylated-Akt (p-Akt) by the Western blotting technique as described earlier.

#### Roles of NOS and HO pathways in the nicotine-acetylcholine interaction

Two sets of experiments were performed. In the first set, 6 groups of sham rats were used (4 nicotine-treated and 3 saline-treated, n = 6–8 each) to test the effect of inhibition of NOS (L-NAME) or HO (ZnPP) or HO induction (hemin) on acetylcholine vasodilations. On day 14 of nicotine (1 mg/kg/day i.p.) or saline treatment, kidneys were isolated, perfused, and preconstricted with phenylephrine. Dose-response curves for acetylcholine (0.01–2.43 nmol) were established 20 min after the infusion of of L-NAME (100 µM), ZnPP (1 µM), or hemin (100 µM). One more group of nicotine-treated rats was used to test the effect of the phosphatidylinositol 3-kinase (PI3K) inhibition by wortmannin 100 nM [31] on acetylcholine vasodilations.

In the second set, two groups of nicotine-treated sham rats were used (n = 6–8 each) to test whether HO and NOS pathways crosstalk in modulating the nicotine-acetylcholine interaction. In these preparations, acetylcholine responses were established 20-min after the infusion of ZnPP + L-arginine (NOS substrate, 100 µM), or L-NAME + hemin (HO inducer, 100 µM).

#### Modulation by ovarian hormones of the nicotine-acetylcholine interaction

This experiment investigated the effects of withdrawal or replacement with ovarian hormones on the interaction of nicotine with acetylcholine vasodilations. A total of 8 groups of female rats (n = 6–8 each) were employed (2 OVX, 2 OVX/E2, 2 OVX/MPA, and 2 OVX/E2/MPA). One group from each category received nicotine (1 mg/kg/day i.p.) for 14 consecutive days and the other group received equal volume of saline. A daily s.c. dose of E2 (50 µg/kg) or MPA (10 mg/kg) was given daily for 5 consecutive days starting from the 9^th^ day after OVX [12], [23] The last dose of E2 or MPA was given 24 hr prior to experimentation. On day 14, the kidneys were isolated, perfused, and preconstricted with phenylephrine. Dose- response curves for acetylcholine (0.01–2.43 nmol) were established as detailed above.

#### The mechanism of the MPA-evoked inhibition of acetylcholine responses in OVX rats

The results of the preceding experiment showed that supplementation of OVX rats with MPA attenuated acetylcholine vasodilations. In this experiment, one group of OVX/MPA rats was employed to test whether this effect of MPA is mediated via progesterone receptors. Each rat received 2 doses of mifepristone (progesterone receptor antagonist, 10 mg/kg s.c.) on the 9th and 12th days after OVX [32]. Mifepristone injections were made 1 hr prior to MPA administration. On day 14, the kidneys were isolated and preconstricted with phenylephrine and acetylcholine Dose-vasodilatory response curves were established. Another two groups of OVX/MPA rats were employed to investigate whether increased generation of NO or CO could compromise the inhibitory effect of MPA on acetylcholine vasodilations. Kidneys obtained from OVX/MPA rats were infused with L-arginine (NOS substrate, 100 µM) or hemin (HO inducer, 100 µM) [33]. Twenty min later, acetylcholine responses were established.

### Statistics

Values are expressed as means±S.E.M. Vasodilatory responses were expressed as a percent of phenylephrine preconstriction. As a measure of the cumulative vasodilatory response, areas under the curve (AUC) of the entire vasodilatory-response curves were computed using trapezoidal integration with zero line taken as the baseline (Graph pad prism, version 3.02) [11], [12]. The repeated measures ANOVA was used for analysis of the acetylcholine dose-response curves. The one-way ANOVA was used for comparing the AUCs. The Bonferroni test was used for the post-hoc analysis with the level of significance set at P<0.05.

## Results

### Nicotine increases acetylcholine, but not NECA or papaverine, vasodilations

Under a constant flow rate of 5 ml/min, the average basal renal perfusion pressure in isolated perfused kidneys obtained from sham rats treated with nicotine 0.5 mg/kg/day (120.4±4.4 mmHg), nicotine 1 mg/kg/day (103.4±9.9 mmHg), nicotine 2 mg/kg/day (86.3±7.9 mmHg), or nicotine 4 mg/kg/day (78.3±6.2 mmHg) were not statistically different from that of saline-treated rats (94.4±7.7 mmHg). Also, the elevations in the renal perfusion pressure caused by continuous infusion of phenylephrine averaged 107.4±10.4 mmHg, and were similar in perfused kidneys obtained from nicotine or saline-treated groups.

**Figure 1:**
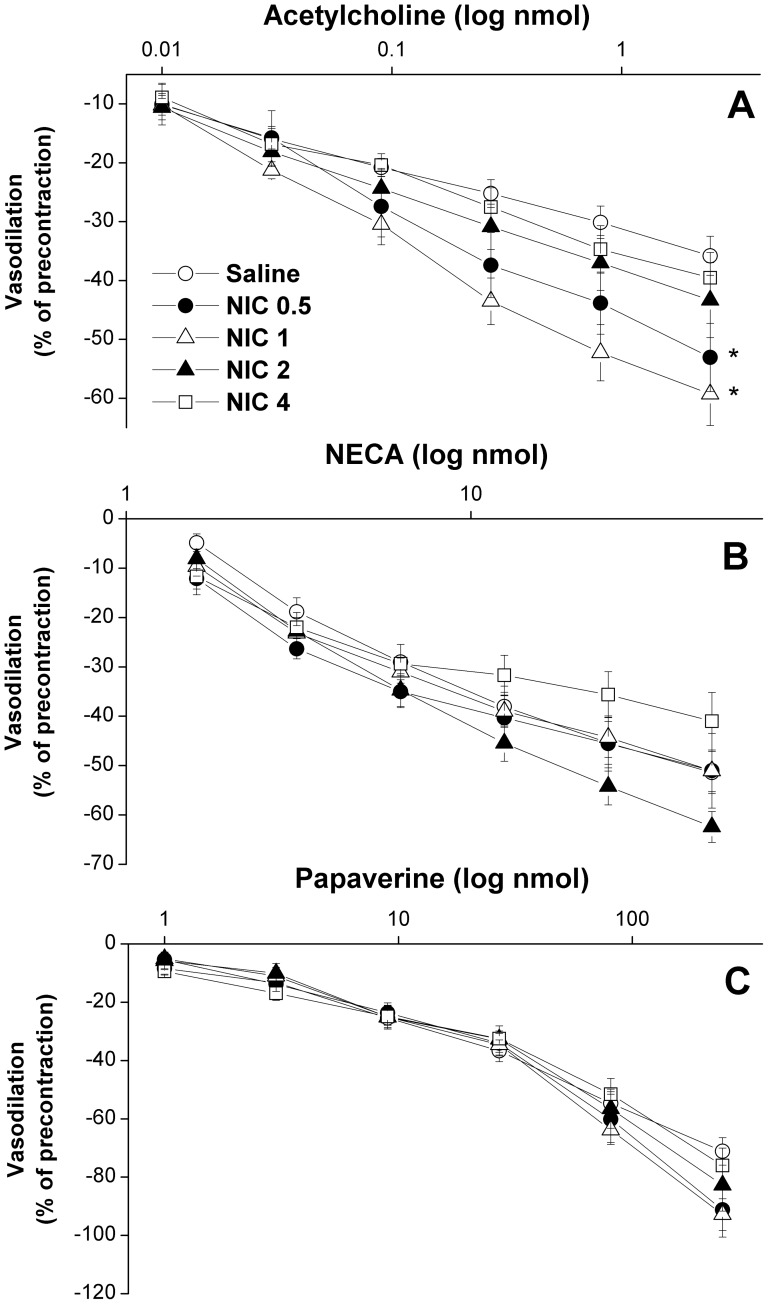
Effect of nicotine (0.5–4 mg/kg/day, 2 weeks) on cumulative vasodilatory effects of acetylcholine (0.01–2.43 nmol), NECA (1.6–50 nmol), and papaverine (1–2.43 nmol) in phenylephrine (10 µM)-preconstricted isolated perfused kidneys obtained from sham rats. Vasodilator responses are expressed as percentages of the phenylephrine-induced tone. Values are means±S.E.M. of 6–8 observations. ^*^P<0.05 vs. saline values.

**Figure 2:**
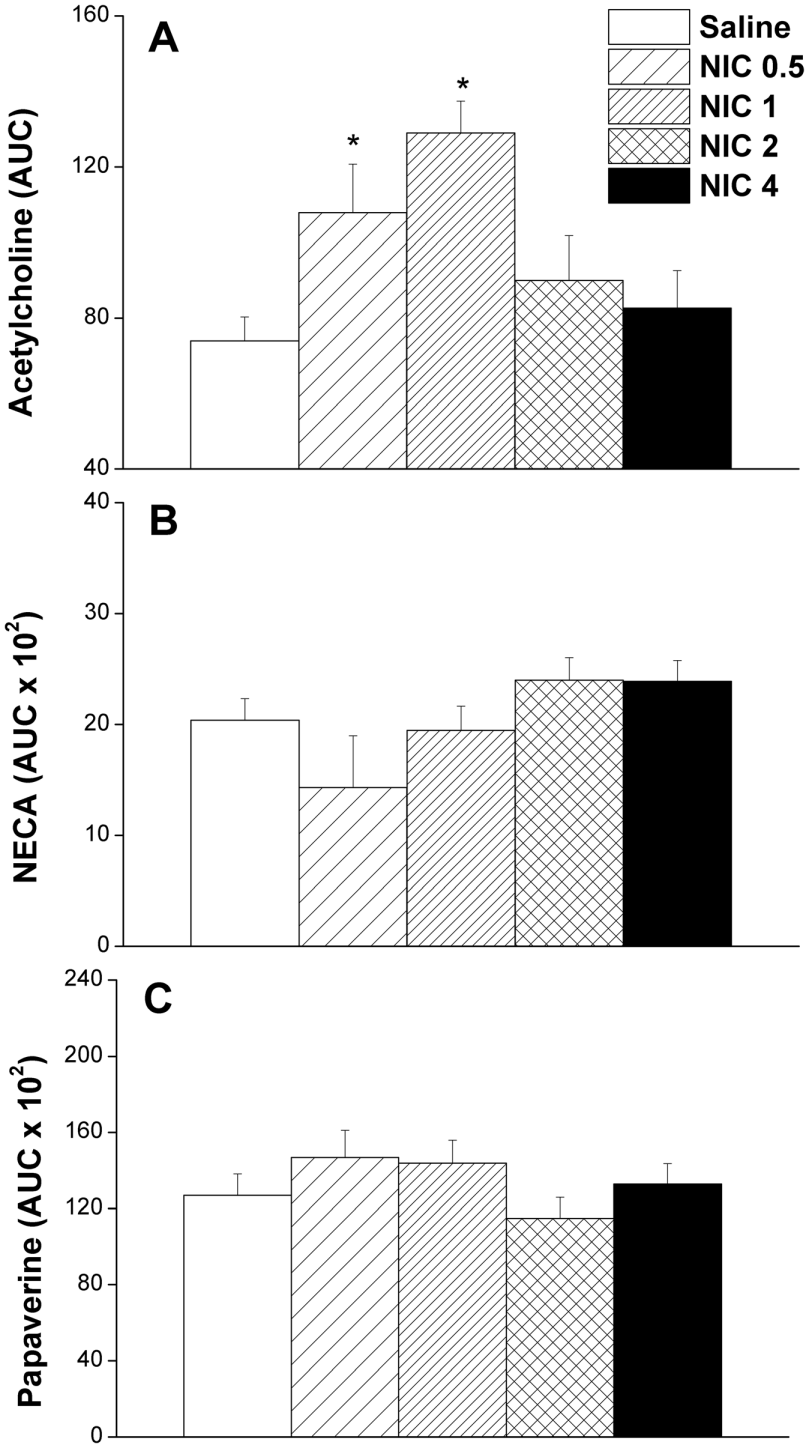
Effect of nicotine (0.5–4 mg/kg/day, 2 weeks) on areas under the curves (AUC, %vasodilation.nmol) of the cumulative renal vasodilatory effect of acetylcholine (0.01–2.43 nmol), NECA (1.6–50 nmol), and papaverine (1–2.43 nmol) in phenylephrine (10 µM)-preconstricted isolated perfused kidneys obtained from sham rats. Values are means±S.E.M. of 6–8 observations. ^*^P<0.05 vs. saline values.

Figures 1 and 2 illustrate the effect of different dose regimens of nicotine on renal vasodilations caused by acetylcholine, NECA, or papaverine in perfused kidneys obtained from sham rats. The cumulative vasodilatory responses caused by acetylcholine (0.01–2.43 nmol) were significantly and dose-dependently increased in preparations treated with nicotine 0.5 or nicotine 1 mg/kg/day compared with saline-treated values (Fig. 1A). In contrast, the higher doses of nicotine (2 or 4 mg/kg/day) failed to affect acetylcholine renal vasodilations (Fig. 1A). Computation of the AUC of the cumulative vasodilatory action during the entire dose-response curve also showed significant increases in acetylcholine vasodilations in perfused kidneys of rats treated with the two lower doses of nicotine (0.5 and 1 mg/kg/day, Fig. 2A). On the other hand, renal vasodilations caused by NECA (1.6–50 nmol, Figs. 1B, 2B) or papaverine (1–243 nmol, Fig. 1C, 2C) were not affected by any of the tested nicotine regimens.

### NOS and HO pathways mediate the facilitation of acetylcholine vasodilations by nicotine

**Figure 3:**
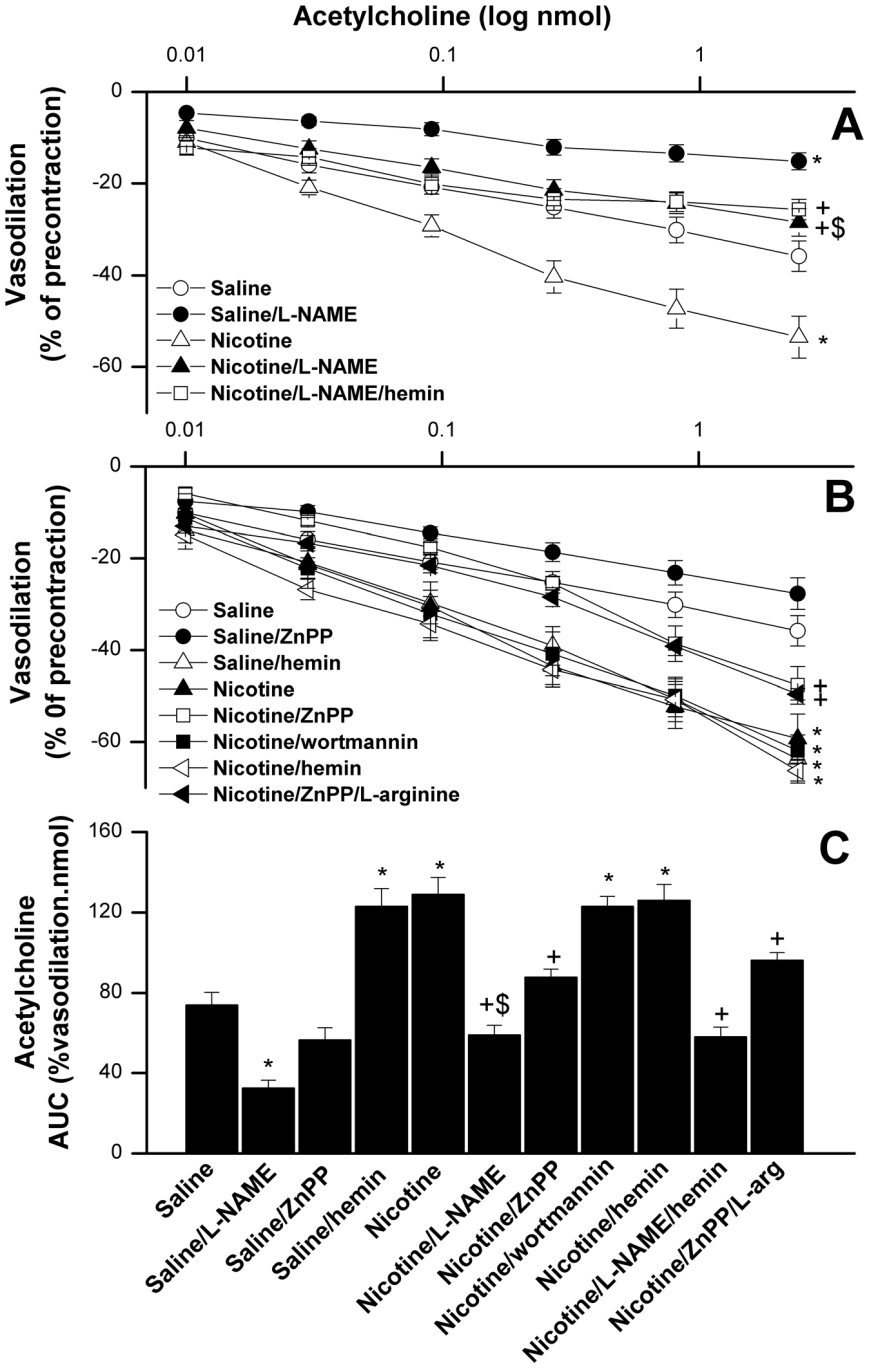
The effects of inhibition of NOS (L-NAME, 100 µM, panel A) and HO (ZnPP, 1 µM, panel B) on vasodilatory effects of acetylcholine (0.01–2.43 nmol) in phenylephrine (10 µM)-preconstricted isolated perfused kidneys obtained from sham rats treated with nicotine (1 mg/kg/day, 2 weeks) or equal volume of saline. The effects of L-arginine (NOS substrate, 100 µM), hemin (HO inducer, 100 µM), or wortmannin (100 nM) are also shown. Panel C shows areas under the curves (AUC, %vasodilation.nmol) of the acetylcholine vasodilatory response. Values are means±S.E.M. of 6–8 observations. ^*^P<0.05 vs. saline values, ^+^P<0.05 vs. nicotine values, ^$^P<0.05 vs. saline/L-NAME values.

The effects of the inhibition of NOS (L-NAME) or HO (ZnPP) on acetylcholine vasodilations in phenylephrine-preconstricted kidneys obtained from sham rats treated with or without nicotine (1 mg/kg) are depicted in figure 3. The infusion of L-NAME (100 µM) into the renal vasculature significantly reduced the vasodilatory effect of acetylcholine (Fig. 3A) and the AUC (Fig. 3C) in sham preparations treated with or without nicotine. The attenuation by L-NAME of acetylcholine vasodilations seen in nicotine-treated sham preparations was preserved in preparations infused simultaneously with hemin (HO inducer, 100 µM).

**Figure 4:**
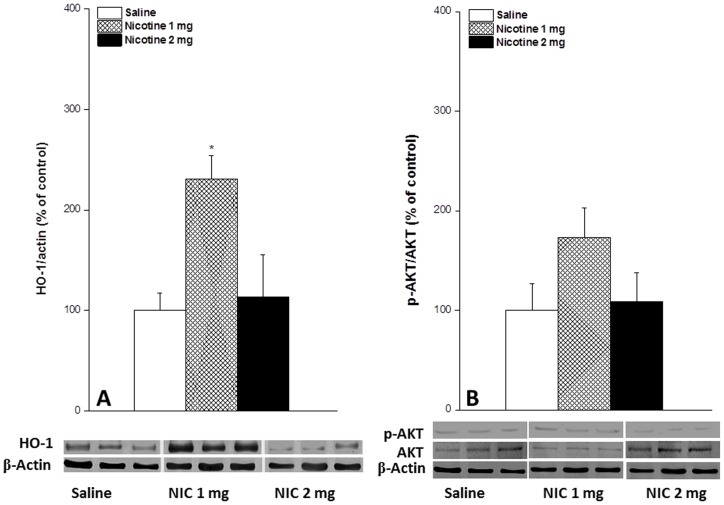
Effect of nicotine (1 or 2 mg/kg/day, 2 weeks) on the protein expression of HO-1 (panel A) and p-Akt protein expression (panel B) in renal tissues of sham rats. Illustrative gels depicting renal HO-1 and p-AKT protein expressions are also shown.Values are means±S.E.M. of 6–8 observations. ^*^P<0.05 vs. saline values.

On the other hand, acetylcholine vasodilations were decreased after the inhibition of HO activity by ZnPP (1 µM) but the reductions were not statistically significant when compared with respective saline values (Fig. 3B, C). In contrast, the infusion of ZnPP significantly reduced the potentiated acetylcholine responses in nicotine-treated preparations whereas the inhibition of PI3K by wortmannin (100 nM) was without effect (Fig. 3B, C). The vasodilatory responses elicited by acetylcholine in nicotine/ZnPP-treated rats were similar to those of saline-treated rats (Fig. 3B, C). The treatment of saline-treated rats with hemin increased acetylcholine vasosdilations to levels similar to those caused by nicotine. No more increases in acetylcholine responses were seen in nicotine/hemin-treated preparations (Fig. 3B, C). Further, L-arginine (NOS substrate, 100 µM) failed to reverse the ZnPP-evoked reductions in acetylcholine vasodilations in nicotine-treated preparations (Fig. 3B, C). Densitometric analysis of the Western bands showed that the renal HO-1 expression was significantly increased in female rats receiving the 1 mg/kg dose of nicotine in contrast to no effect for the 2 mg/kg/day dose (Fig. 4A). The protein expression of renal p-Akt was slightly increased by the 1 mg/kg/day dose of nicotine compared with saline-treated values but differences were not statistically significant (Fig. 4B).

### Ovarian hormones uncover the nicotine-evoked facilitation of acetylcholine vasodilations

**Figure 5:**
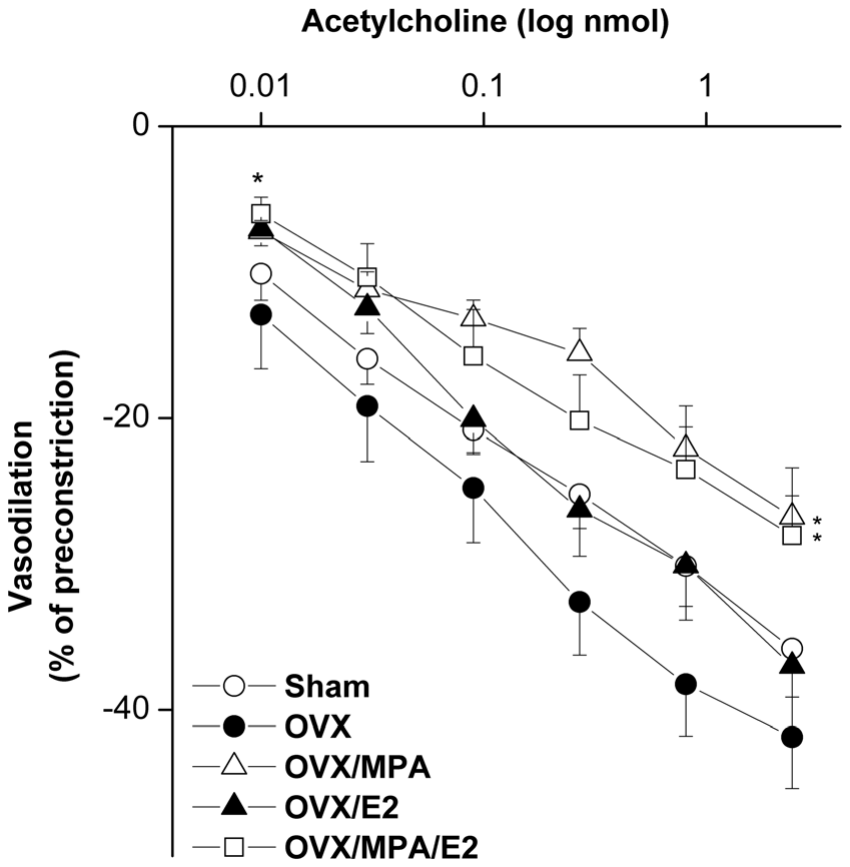
Cumulative vasodilatory effects of acetylcholine (0.01–2.43 nmol) in phenylephrine (10 µM)-preconstricted isolated perfused kidneys obtained from sham, ovariectomized (OVX) rats supplemented with estrogen (OVX/E2), medroxyprogesterone acetate (OVX/MPA), their combination (OVX/E2/MPA), or the vehicle. Acetylcholine responses are expressed as percentages of the phenylephrine-induced tone. Values are means±S.E.M. of 6–8 observations. ^*^P<0.05 vs. OVX values.

**Figure 6:**
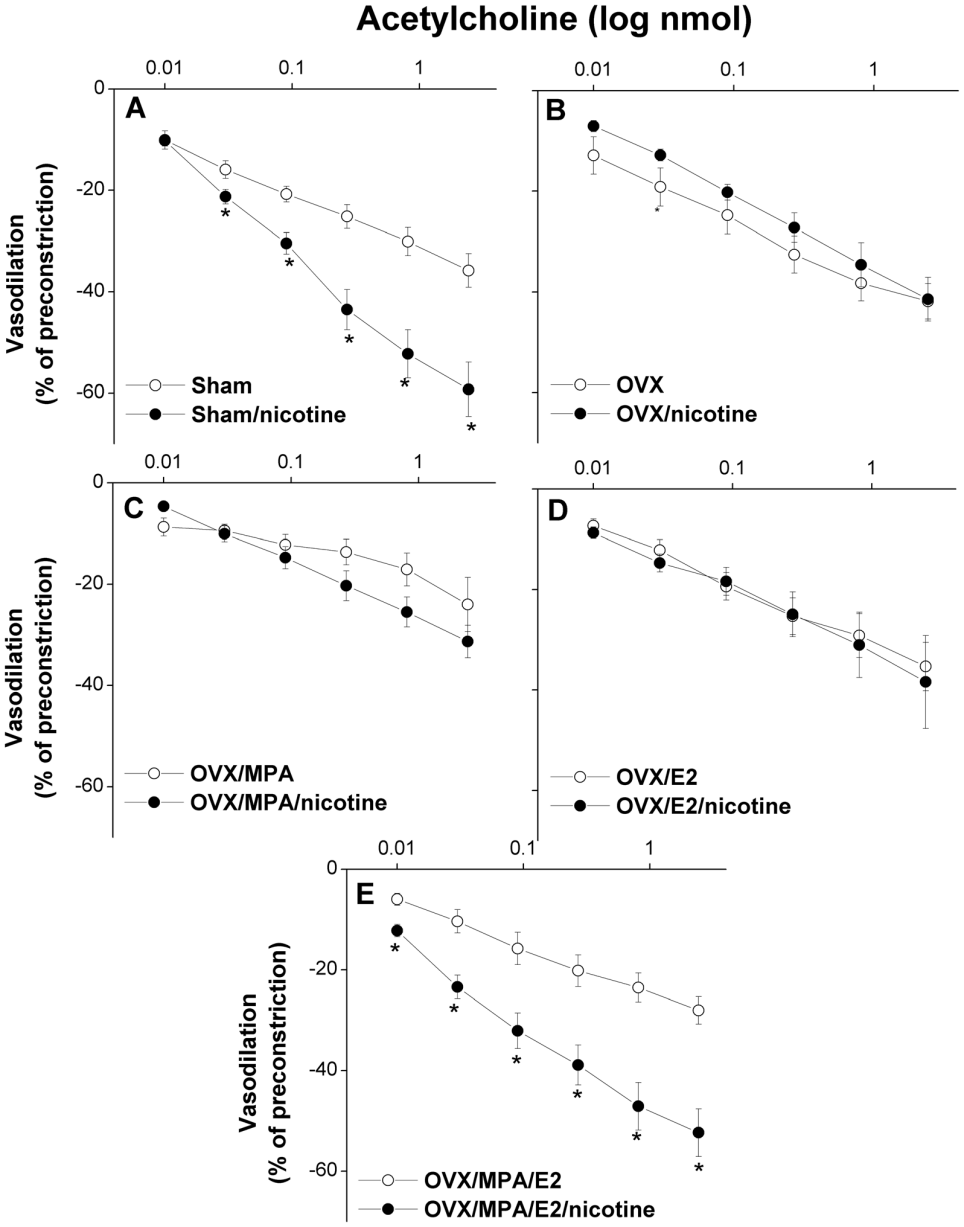
Effect of nicotine (1 mg/kg/day, 2 weeks) on vasodilatory effects of acetylcholine (0.01–2.43 nmol) in phenylephrine (10 µM)-preconstricted isolated perfused kidneys obtained from sham, or ovariectomized (OVX) rats supplemented with estrogen (OVX/E2), medroxyprogesterone acetate (OVX/MPA), their combination (OVX/E2/MPA), or the vehicle. Values are means±S.E.M. of 6–8 observations. ^*^P<0.05 vs. corresponding control values.

**Figure 7:**
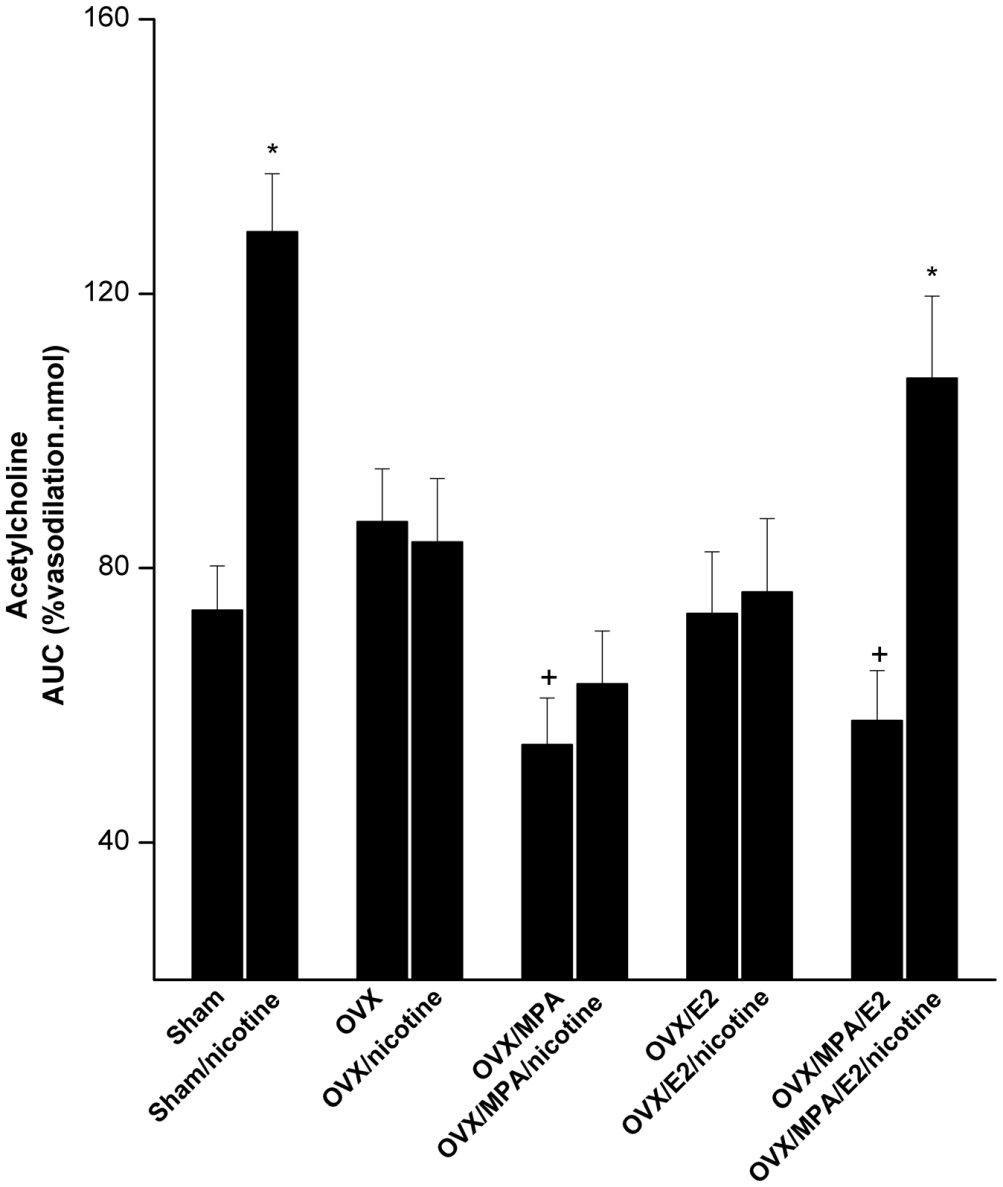
Effect of nicotine (1 mg/kg/day, 2 weeks) on areas under the curves (AUC, %vasodilation.nmol) of the cumulative renal vasodilatory effect of acetylcholine (0.01–2.43 nmol) in phenylephrine (10 µM)-preconstricted isolated perfused kidneys obtained from sham or ovariectomized (OVX) rats supplemented with estrogen (OVX/E2), medroxyprogesterone acetate (OVX/MPA), their combination (OVX/E2/MPA), the vehicle. Values are means±S.E.M. of 6–8 observations. ^*^P<0.05 vs. corresponding control values, ^+^P<0.05 vs. OVX values.

The average basal renal perfusion pressures in perfused kidneys obtained from sham-operated, OVX, OVX/E2, OVX/MPA, and OVX/E2/MPA rats were similar and amounted to 94.4±7.7, 91.8±4.2, 116.9±6.8, 103.4±5.9, and 100.2±5.5 mmHg, respectively. Also, the elevations in the renal perfusion pressure caused by continuous infusion of phenylephrine in these preparations were not statistically different (data not shown). Figures 5–7 illustrate the effects of OVX and hormone replacement regimens on renal vasodilations caused by acetylcholine in the absence and presence of nicotine (1 mg/kg for 2 weeks). Compared with sham values, the cumulative vasodilatory responses caused by acetylcholine (0.01–2.43 nmol) were not affected by OVX or E2 (50 µg/kg/day s.c.) supplementation to OVX rats (Fig. 5 and 7). Hormone replacement of OVX rats with MPA (10 mg/kg/day s.c.) or MPA+E2 significantly attenuated acetylcholine responses compared to OVX values (Fig. 5 and 7).

Compared with saline-treated preparations, nicotine (1 mg/kg/day for 2 weeks) increased the vasodilatory effect of acetylcholine in perfused kidneys obtained from sham and OVX/E2/MPA rats (Figs. 6 and 7). In preparations obtained from OVX, OVX/E2, or OVX/MAP rats, similar vasodilatory responses for acetylcholine were demonstrated in nicotine and saline-treated groups (Figs. 6 and 7).

### Hemin reverses the inhibitory effect of MPA on acetylcholine vasodilations

**Figure 8:**
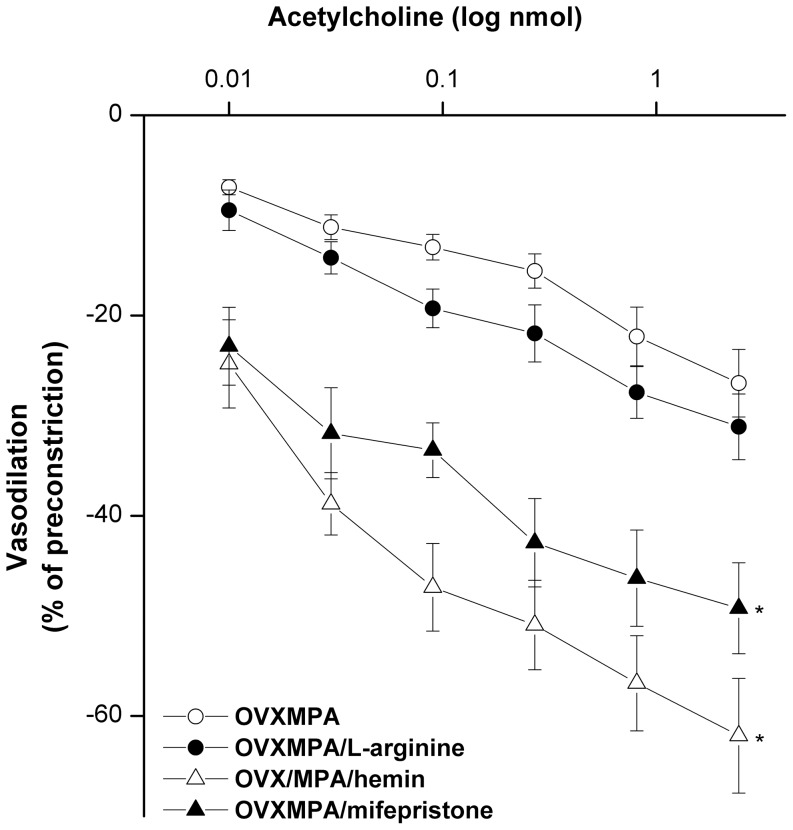
Effects of L-arginine (NOS substrate), hemin (HO inducer) or mifepristone (progesterone receptor blocker) on the vasodilatory action of acetylcholine (0.01–2.43 nmol) in phenylephrine (10 µM)-preconstricted isolated perfused kidneys obtained from OVX/MPA rats. Values are means±S.E.M. of 6–8 observations. ^*^P<0.05 vs. OVX/MPA values.

The reduced acetylcholine vasodilations caused by MPA supplementation to OVX rats were reversed when OVX/MPA rats were treated concurrently with mifepristone (progesterone receptor blocker, 10 mg/kg s.c., Fig 8). Similar increases in the vasodilatory responses to acetylcholine were observed when OVX/MPA kidneys were infused with hemin (HO inducer, 1 µM) but not L-arginine (NOS substrate, 100 µM) (Fig. 8).

## Discussion

The current study attempted to investigate the dose, NOS/HO, and hormonal dependencies of the interaction of chronic nicotine with the renal vasodilator capacity in female rats. The most important findings of the present study can be summarized as follows. First, the 2-week treatment with relatively low doses of nicotine (0.5 and 1 mg/kg/day) caused dose-dependent increases in cholinergic, but not adenosinergic or papaverine, renal vasodilations Second, the upregulation of the PI3K/Akt-independent NOS/HO signaling mediates the facilitatory effect of nicotine on acetylcholine vasodilations because (i) the potentiated acetylcholine responses in nicotine-treated preparations were significantly reduced after selective inhibition of HO-1 (ZnPP) or NOS (L-NAME), but remained unaltered in tissues treated with the PI3K inhibitor wortmannin, (ii) the ZnPP effect was not altered after co-infusion of L-arginine, and (iii) nicotine increased the protein expression of renal HO-1 but not p-Akt. Third, the presence of the two ovarian hormones, estrogen and progestin, is mandatory for the facilitated cholinergic activity evoked by nicotine because the latter disappeared in OVX rats treated with or without mono-hormonal therapies (E2 or MPA) and was restored in OVX supplemented with the dual (E2/MPA) hormonal regimen. Fourth, MPA supplementation of OVX rats significantly reduced acetylcholine responses through a mechanism that was progesterone receptor-sensitive and was reversed in prepartions treated with the HO inducer hemin.

Consistent with the multiple and divergent vascular effects of nicotine [11], [13], [17], [20], the present study demonstrated that the effect of chronic nicotine on the renal vasodilator capacity depended primarily on the dose of nicotine and the mechanism of the vasodilator stimulus. Nicotine increased cholinergically-mediated renal vasodilations in contrast to no effect on vasodilations caused by the adenosine analogue NECA or by the phosphodiesterase inhibitor papaverine. This discrepany infers differences in the way by which nicotine interacts with cellular sites provoking renal vasodilations or respective downstream signaling molecules. Moreover, the facilitatory effect of nicotine on acetylcholine renal vasodilations was dose specific as it was evident in rats receiving the low (0.5 and 1 mg/kg/day) and not the high (2 and 4 mg/kg/day) doses of nicotine. Remarkably, our previous studies showed that measurement of plasma cotinine, the principal metabolite and pharmacologically active form of nicotine, in female rats receiving similar doses of nicotine [34] were comparable to levels achieved in humans after moderate cigarette smoking [35], [36], which establishes the clinical relevance of the current findings.

The gaseous molecules NO and CO, products of NOS (L-arginine) and HO (heme) enzymatic activities, respectively, are important modulators of vascular control in renal and extra-renal vascular beds [8], [10], [37], [38]. Moreover, an interplay has been established between NO and CO in vascular control [39]–[41]. The current findings in perfused kidneys of control (saline-treated) rats that acetylcholine vasodilations were reduced after the inhibition of NOS (L-NAME) but not HO (ZnPP) suggest preferential involvement of NOS-derived NO in cholinergically-mediated vasodilations. By contrast, the facilitation of both NOS/NO and HO/CO signaling appears to mediate the nicotine-evoked facilitation of acetylcholine vasodilations. This conclusion is bolstered by the observation that the inhibition of NO (L-NAME) or CO (ZnPP) synthesis significantly reduced the potentiated acetylcholine responses in nicotine-treated preparations. Because the effect of ZnPP was not reversed after supplementation with L-arginine, the possibility of CO/NO crosstalk in modulating the nicotine- acetylcholine interaction is unlikely.

Another important proof for the involvement of the enzymatic products of HO-1 in the facilitated acetylcholine vasodilation caused by nicotine emerged from the protein expression studies. Densitometric analysis of the Western bands revealed that the dose specificity of the nicotine effect on renal HO-1 expression paralleled its interaction with acetylcholine vasodilations. Whereas the enhanced acetylcholine vasodilations caused by the 1 mg/kg/day dose of nicotine was accompanied by increased renal HO-1 protein expression, neither events (acetylcholine response or HO-1 abundance) was altered by the 2 mg/kg/day dose of nicotine. With that said, the increased HO-1 expression does not appear to be mediated via Akt activation (phosphorylation). Indeed, the p-Akt protein expression in renal tissues of nicotine-treated rats was not statistically different from control values. This view is bolstered by the observation that the facilitatory effect of nicotine on acetylcholine vasodilations remained manifest after pharmacologic inhibition of PI3K by wortmannin. Our data, therefore, argue against the involvement of the PI3K/Akt signaling in the cascade of cellular events leading to the HO-1-mediated increases in acetylcholine vasodilations in nicotine-treated rats. Notably, some, but not all, reported studies implicated Akt in the biological effects of HO-1 [42]–[44]. Together, the current findings establish the first pharmacological and molecular evidence that favors a key role for Akt-independent HO-1 upregulation in the facilitatory effect of nicotine on the renal vasodilatroy action of acetylcholine in female rats.

The renovascular effects of nicotine are believed to be modulated by ovarian hormones. For instance, in a recent study [12], we demonstrated that the NOS-dependent vasodilations caused by acute nicotine infusion in perfused kidneys of female rats were reduced in OVX preparations and restored back to control levels after estrogen supplementation. It is concluded that the estrogen-dependent facilitation of NOS signaling mediates the enhanced vasodilator capacity of nicotine in the renal vasculature of female rats [12]. Therefore, in the present study we asked if the altered acetylcholine responsiveness by chronic nicotine in the female renal vasculature is hormonally-dependent. The results showed that the nicotine/acetylcholine renovascular interaction depended fundamentally on the hormonal status because (i) like sham rats, chronic nicotine increased acetylcholine renal vasodilations in E2/MPA-supplemented OVX preparations, and (ii) nicotine caused no changes in acetylcholine responses in OVX treated with or without monohormonal therapies (OVX/E2 or OVX/MPA). It is conceivable, therefore, that the co-existence of the two ovarian hormones is necessary for the demonstration of the facilitatory effect of nicotine on acetylcholine vasodilations. Remarkably, the current study is the first to report on the role of the hormonal state in the renovascular interaction of chronic nicotine with cholinergic vasodilations in female rats. That said, it is not clear whether changes in the hormonally-dependent nicotine-acetylcholine renal interaction are paralleled by similar changes in the Akt/HO-1 signaling. This interesting possibility will be addressed in future studies.

Apart from nicotine, it is imperative to comment on the effect of OVX and hormonal replacement on acetylcholine vasodilations. We showed that the vasodilatory action of acetylcholine was maintained in kidneys obtained from OVX rats, a model of surgical menopause [45], or E2-replaced OVX rats (Fig. 5), suggesting no tonic modulatory effects for E2 on acetylcholine vasodilations. On the other hand, the vasodilatory action of acetylcholine was significantly reduced in OVX rats treated with MPA alone or combined with E2, thereby suggesting an inhibitory effect for MPA on acetylcholine responses. Because the inhibitory action of MPA was virtually abolished after blockade of progesterone receptors (mifepristone) and in preparations with facilitated HO (hemin), but not NOS (L-arginine) activity, it is possible that the inhibition of HO activity next to the activation of progesterone receptors might account for the MPA-evoked attenuation of acetylcholine vasodilations. A similar impairment by MPA of endothelial function in the brachial artery of young women has been reported [46]. In support of this presumed inverse relationship between progesterone receptors and HO-1 activity, Cable et al. [47] demonstrated that the treatment of cultured chick embryo liver cells with mifepristone, a selective progesterone receptor blocker, increases HO activity. Further, reciprocal changes in HO-1 expression (increases) and serum progesterone (decreases) have been shown to associate the antitumor activity of melatonin in the rat mammary carcinoma model [48] and the high glucose induced steroidogenesis in adrenal cells [49]. Notably, the doses of E2 or MPA employed in the present study have been used in previous studies including ours and found to produce physiological levels of the hormones [12], [50].

One potential limitation for the HO inhibitor ZnPP relates to its ability to inhibit other enzymatic systems such as NOS and sGC [51], [52]. Ny et al. [53] reported that ZnPP reduces acetylcholine relaxations in the rat aorta via interfering with HO-independent membrane receptor-coupled signal transduction pathways. Paradoxically, ZnPP can also induce HO-1 in some settings such as ischemia [54]. A modulatory effect for ZnPP on cytochrome P-450 has also been described [55]. The interaction of ZnPP with NOS is particularly important given the scope of the current research. Our findings that L-NAME, but not ZnPP, reduced acetylcholine vasodilations in intact female kidneys argue against a possible NOS inhibitory effect for ZnPP. Also, the concentration of ZnPP (1 µM) employed in the current study has been shown to exerts greater inhibitory effect on HO compared with NOS [56].

The current finding that NECA vasodilations were not affected by nicotine is consistent with a recent study from our laboratory [23]. Nonetheless, comparisons of current and previous data [23] revealed that hormonal modulation of the nicotine interaction with the vasodilatory response depends primarily on the receptor site that elicits the response. For instance, E2 or MPA replacement of OVX rats increased NECA vasodilations [23] in contrast to no effect on acetylcholine vasodilations (this study). Also, nicotine potentiated acetylcholine (this study), but not NECA [23], vasodilations in female preparations with balanced hormonal profile (e.g. sham or OVX/E2/MPA rats). Although the reason for the discrepancy in the acetylcholine and NECA profiles is not clear, the current and previous data [23] obviously reflect some differences in the hormonal/nicotine interaction with cellular sites that provoke the renal vasodilatory response or perhaps their downstream effectors. More studies are needed to validate this possibility.

The clinical significance of the present investigation is two-fold. First, the capacity of nicotine to facilitate the renal cholinergic vasodilatory activity together with the reported NOS-dependent vasodilatory action of nicotine in the renal vasculature [12] might infer a therapeutic benefit for nicotine in pathological conditions for which endothelium dysfunction might be a predisposing factor. This view is supported by other reports in which nicotine favorably impacts proteinuric and inflammatory kidney diseases [57]. The second clinically important observation pertains to the finding that hemin rectified the negative impact of MPA on renovascular responsiveness to acetylcholine, thus reflecting a fundamental renovascular protective role for hemin in females receiving MPA. This is consistent with published data concerning the ability of hemin to prevent the rejection of kidney allografts in rats [58], reduce the progression of renal fibrosis [59], and attenuate polymyxin B-induced nephrotoxicity [60].

Collectively, the present study provides important insights into the chronic effect of nicotine on the renal vasodilator propensity in female rats. Nicotine at relatively moderate exposure levels enhanced the vasodilator responsiveness to cholinergic activation. The most prominent features of the facilitatory effect of nicotine are: (i) its dependence on the nicotine dose, (ii) the underlying mechanism involves the activation of PI3K/Akt-independent NOS/HO-1 signaling, and (iii) it is highly reliant on the specific hormonal setting, being manifest only in female preparations with balanced E2/progestin milieu. Clinically, nicotine supplements might be exploited to rectify pathological conditions characterized of having impaired vascular endothelial activity.
